# Coping and Quality of Life Differences between Emergency and Rehabilitation Healthcare Workers

**DOI:** 10.3390/healthcare11162235

**Published:** 2023-08-09

**Authors:** Davide Cardile, Francesco Corallo, Augusto Ielo, Irene Cappadona, Maria Pagano, Placido Bramanti, Giangaetano D’Aleo, Rosella Ciurleo, Maria Cristina De Cola

**Affiliations:** 1 IRCCS Centro Neurolesi Bonino-Pulejo, S.S. 113 Via Palermo, C.da Casazza, 98124 Messina, Italy; davide.cardile@irccsme.it (D.C.); augusto.ielo@irccsme.it (A.I.); irene.cappadona@irccsme.it (I.C.); maria.pagano@irccsme.it (M.P.); placido.bramanti@irccsme.it (P.B.); giangartano.daleo@irccsme.it (G.D.); rossella.ciurleo@irccsme.it (R.C.); mariacristina.decola@irccsme.it (M.C.D.C.); 2 Faculty of Psychology, Università degli Studi eCampus, Via Isimbardi 10, 22060 Novedrate, Italy

**Keywords:** EASY-NET, audit, quality of life, burnout, emergency, rehabilitation

## Abstract

Audit and Feedback (A&F) is a systematic process involving the collection of data, which are subsequently compared with the established reference standards and then subsequently disseminated to healthcare providers through feedback meetings. This allows continuous improvement to be ensured in the quality of care processes. Often, the parameters taken into account concern only the patient and the treatment processes, neglecting other variables. Quality of life in the workplace and coping skills are determining variables for the clinical performance of all healthcare professionals. For this reason, in this study, these variables were investigated and differences were highlighted in two different role categories and context: cardiovascular emergency and neurological rehabilitation. A psychological screening was carried out by sending the computerized Coping Orientation to Problems Experienced—Nuova Versione Italiana (COPE—NVI) and Professional Quality of Life Scale—5 (ProQoL) questionnaires to all healthcare workers involved. Ninety-five healthcare providers (mean ± SD age: 47 ± 10.4 years; 37.9% male) answered the questionnaire and were assigned into two groups (G1 and G2) based on the ward in which they worked. These were further divided into two subgroups (R1 and R2) based on their role. The obtained results show that avoidance strategies are used more by health professionals working in rehabilitation (G2) wards than in intensive-care units (G1). Moreover, in G1 nurses, physical therapists and speech therapists (R2) obtained higher scores in terms of turning to religion (TR) and compassion satisfaction (CS), while physicians and psychologists (R1) obtained higher scores on the burnout scale (BO). The TR score for R2 was found to be higher, even in G2. The response trend of the two groups in the different departments was analyzed and commented on.

## 1. EASY-NET Project, Coping, and Quality of Life

The term Audit & Feedback (A&F) refers to a systematic process of collecting data, which are subsequently compared with established reference standards and then provided to healthcare providers through feedback meetings [1,2]. To assess the effectiveness of A&F strategies and to enhance clinical practice and equity in various settings, the EASY-NET project was initiated [3]. This Italian network project, funded by the Italian Ministry of Health and launched on 15 April 2019, aims to evaluate different aspects related to both patients and the care process. Patient-related aspects encompass the process of care, effectiveness, clinical outcomes, and safety, while the aspects of medical performance include equity, efficiency, and timeliness [4,5]. The EASY-NET project encompasses seven Italian regions: Lazio, Friuli Venezia Giulia, Piedmont, Emilia-Romagna, Lombardy, Calabria, and Sicily. Each region corresponds to a specific Work Package (WP 1–7) outlining their respective areas of intervention within the EASY NET project. WP7, led by IRCCS Centro Neurolesi Bonino Pulejo in Sicily, focuses on acute myocardial infarction (AMI) and ischemic stroke (IS) settings, covering both the acute and rehabilitation phases. Its primary objective is to assess the aforementioned aspects to enhance the quality, safety, and accessibility of healthcare processes and services [6].

The innovation introduced by WP7 involves integrating the traditional A&F model with telemedicine protocols and addressing the psychological variables of healthcare operators working in the context of cardio- and cerebrovascular emergencies (AMI and ischemic stroke), as well as neurological rehabilitation (ischemic stroke). Psychological and emotional variables significantly influence an individual’s well-being, work performance, and, ultimately, patient well-being [7,8,9]. As part of this study, healthcare workers within the sample underwent psychological screening to assess coping skills and quality of life in the workplace.

The term “coping” refers to the methods of coping with, reacting to, resisting, and managing stress [10,11]. Stress is the outcome of an appraisal process that emerges when external (environmental) and/or internal (personal) demands put an individual’s resources to the test [12]. In fact, people interpret events in an immediate way, and it is mainly this cognitive evaluation of the event, rather than the event itself, that triggers or does not trigger a reaction [13]. There are two types of appraisals: primary and secondary [14]. Primary appraisal is the initial assessment of the significance of an event, which one interprets as more or less relevant depending on one’s own wellbeing and goals. Secondary appraisal, on the other hand, examines the resources and options at one’s disposal and considers how one can face a situation [15].

Initially, coping was conceived as a response to exclusively external stressful or negative events, but subsequently, the idea that coping and stress were as much about the external as the internal world became more established (ibidem). Today, coping is considered an adaptive and dynamic process, as it is expressed in the interaction and reciprocal influence between the individual and the environment [16].

Today, coping is considered to be an adaptive and dynamic process, as it is expressed in the interaction and reciprocal influence between the individual and the environment [17,18]. Coping strategies refer to the ways in which people cope with and manage different situations [19,20]. They can assume functional or dysfunctional valence, and when becoming stable, they characterize the personal style with which people typically cope with situations in everyday life [21,22].

Quality of life in the workplace is identified as the level of satisfaction related to the work performed and the corporate environment [23]. When working in a quality and efficiently organized environment, the sense of security and personal and professional development among employees increases. High satisfaction levels are associated with better work performance [24,25,26]. On the contrary, job dissatisfaction reduces employee engagement and increases turnover rates [27]. The main causes of work-related stress include workload, relationship problems with patients or colleagues, work–life balance, and lack of safety in the workplace [28,29]. Of course, a more or less adequate remuneration also influences the perceived stress level and satisfaction. In addition, healthcare professionals constantly have to deal with the suffering of patients in their daily practice [30,31]. This makes the caring profession highly demanding from an emotional, physical, and spiritual point of view [32]. A perceived negative work environment often leads workers to feel exhausted and to suffer from burnout and other syndromes [33,34]. Burnout constitutes a feeling of exhaustion and failure that results from an overuse of workers’ personal resources or physical, mental, and spiritual energy, preventing them from providing care and assistance to the users of organizations in different sectors whose primary goal is to help others [35,36]. One of the events that has had a significant impact on healthcare workers was the COVID-19 pandemic. During this worldwide emergency, they have been at the forefront of managing the crisis, facing increased workloads, high levels of stress, and a heightened risk of infection, which has caused increased levels of anxiety, depression, insomnia, and distress [37]. To cope with these challenges, healthcare workers have employed various coping strategies, including seeking support from colleagues, friends, and family; engaging in self-care activities; maintaining a positive attitude; and using problem-solving techniques [38]. Among these strategies, the ones that proved to be the most effective during the pandemic were problem-focused coping and seeking social support, because these were associated with better mental health outcomes [39]. Moreover, task-oriented and adaptive coping resulted to be protective factors for burnout, while avoidance-oriented and maladaptive coping were found to be predictive factors of burnout [40]. Other aspects that constituted beneficial or protective factors were compassionate interactions among healthcare professionals, adhering to infection control measures, and maintaining a healthy lifestyle [39,41]. Given the importance of coping strategies and the quality of the working life of healthcare workers [42], WP7 considered these aspects during the development of the A&F intervention. This study describes such assessments conducted on healthcare workers, comparing the emergency setting with the rehabilitative setting, and the role of healthcare within the care pathway. In addition, this paper aims to raise awareness regarding the importance of including assessments of the psychological aspects of healthcare workers in quality control procedures, and more specifically in audit and feedback processes.

## 2. Materials and Methods

The target population for the A&F intervention consisted of healthcare workers in the context of cardio- and cerebrovascular emergency (AMI and ischemic stroke) and in neurological rehabilitation (ischemic stroke). The participants were recruited from Sicilian hospitals included in the EASY-NET project [4]. For the acute setting, eligible hospitals were required to include: (i) an emergency room; (ii) at least a service for the treatment of cardiovascular diseases (coronary units and/or interventional cardiology services); and (iii) at least a service for the treatment of cerebrovascular diseases (stroke unit and/or interventional neuroradiology service). For the neurorehabilitation setting, eligible hospitals were required to possess a neurological rehabilitation unit for stroke survivors. In order to conduct psychological screening on different hospital populations, two questionnaires were computerized and sent to physicians and nurses at all hospitals involved in the study. Since this work did not involve testing or studying a clinical procedure or tool, approval from the ethics committee was not required. However, all participants signed an informed consent form to participate to the study, and the questionnaires were submitted anonymously.

### 2.1. Study Population

Ninety-five healthcare providers (mean ± SD age: 47 ± 10.4 years; 37.9% male) submitted the online survey and were subsequently categorized into two groups (G1: n = 49, G2: n = 46) based on the clinical area and the ward in which they worked (G1 = cardiovascular intensive care unit; G2 = neurological rehabilitation unit). The subjects who participated in the study were further divided into two subgroups based on their roles (management or operational role): the first subgroup (R1 = Role 1) consisted of physicians and psychologists, while the second subgroup (R2 = Role 2) consisted of nurses, physical therapists, and speech therapists. Statistical analysis was also carried out for the subgroups. Table 1 shows the descriptive characteristics of the study population.

### 2.2. Instruments

The screening was aimed at highlighting the strategies used to cope with stressful events and the quality of life of operators in different departments. The questionnaires that were chosen and sent to healthcare providers were as follows:

- Coping Orientation to Problems Experienced—Nuova Versione Italiana (NVI) [43] is a 60-item validated self-report questionnaire that asks the subject to rate how often he or she enacts a particular coping process in difficult or stressful situations. The subject should not refer to a specific stressor, but rather think about how he or she habitually behaves in stressful situations. There are four possibilities for response, from “I usually don’t” to “I almost always do”. From the scores recorded for the different items, it is possible to deduce 5 scales: social support (SS), avoidance strategies (AS), positive attitude (PA), problem solving (PS), and turning to religion (TR). Items on the social support scale refer to seeking understanding, information, and emotional venting, so if the scores obtained turn out to be excessively high, this indicates a certain passivity in the individual’s handling of stress and anxiety. The avoidance strategy scale is very heterogeneous and includes denial use, substance use, and behavioral and mental detachment; again, the higher on the scale, the more the subject will tend to employ strategies aimed at not directly addressing the problem. The positive attitude scale indicates an attitude of acceptance, containment, and positive reinterpretation of events. Obtaining a high score for the items on this scale indicates a positive attitude toward one’s work and activities. The items on the problem-solving scale refer to the use of active strategies and planning, so a high score on this scale suggests that the person is used to dealing directly with problems by stopping to think, planning strategies, and implementing them effectively. Finally, the turning to religion scale items refer to taking refuge in religion and humor, so as with the social support scale, a score is synonymous with a certain passivity in dealing with anxiety and stress.
- The Professional Quality of Life Scale—5 (ProQoL) [44] is a 30-item validated self-report questionnaire aimed at measuring job satisfaction, compassion fatigue, and burnout in workers in the helping profession. It proves to be a useful tool to apply to workers who work in settings affected by high emotional burden where they may be exposed to traumatic events. The ProQoL measure three subscales related to professional quality of life: compassion satisfaction, burnout, and secondary traumatic stress. Compassion satisfaction (CS) corresponds to the satisfaction that is derived from performing one’s work tasks. For example, if a worker derives pleasure from his helping work and feels positively about his colleagues and/or his ability to contribute to the work setting, or even to society, this represents a high level of CS. Burnout (BO) consists of frustration, anger exhaustion, and depression that results from one’s work. It is also accompanied by feelings of hopelessness and difficulty coping or performing effectively. After onset, generally, these negative feelings tend to grow gradually and sometimes reflect the feeling that despite one’s efforts, nothing can change. Sometimes, however, they are associated with very stressful working conditions, such as an unfavorable work environment or a very high workload. The higher the score on this scale, the greater the risk of burnout. Secondary traumatic stress (STS) results in the fear arising from primary or secondary trauma that derives from one’s work. Specifically, it refers to secondary exposure to stressful events that are work-related. The symptoms that result from this exposure, unlike those of burnout, are usually of rapid onset and are associated with a particular event. The obtainable score in each of the three subscales ranges from 0 to 50. For each of the subscales a score of 22 or less is considered as low, a score between 23 and 41 is considered as moderate, and a score of 42 or more is considered as high. By summing the scores obtained on the B scale and the STS scale, it is possible to obtain a score that relates to compassion fatigue, a condition characterized by a gradual, progressive decrease over time in the desire to care, or compassion. This represent a real syndrome that is common among those professionals who work closely with victims of illness, trauma, or disaster, such as the healthcare sector, that arises acutely and suddenly, which can be triggered by even a single experience that is perceived as particularly critical by the person affected.
- The total time to complete the questions included in both questionnaires was approximately 10 min. Following the guidelines, after analyzing and processing the results, they were reported to the different departments in the various facilities through a feedback phase. The latter consisted of the sharing of periodic reporting and/or the provision of meetings to discuss the results of the proposed screening. Obviously, not only the results of the psychological screening were reported, but those of the indicators measured during the audit phase were as well.

### 2.3. Statistical Analysis

All of the analyses were performed using R statistical software, version 4.2.2 [45], considering a *p*-value < 0.05 as statistically significant. The normality of the dependent variables was assessed through the Shapiro–Wilk test, which failed for most of the target variables. Thus, we used a non-parametric approach by assessing the homogeneity of the variances of the two variables through Levene’s test, and then employed the Mann–Whitney U-test to compare the outcomes between the two groups (G1 vs. G2), as well as between the subgroups (R1 vs. R2). The proportions were compared using the Chi-squared test.

## 3. Results

No significant differences in terms of age (*p* = 0.31) between the two groups or subgroups (*p* = 0.24) were found.

Sica et al. [43] stated that a statistical analysis of the COPE-NVI revealed Cronbach’s alpha scores ranging from 0.70 to 0.91. Our study confirmed good internal consistency with the following Cronbach’s alpha scores: social support = 0.83, avoidance strategies = 0.81, positive attitude = 0.76, problem solving = 0.75, and turning to religion = 0.69.

Stamm [44] reported Cronbach’s alpha values of 0.88 for CS, 0.75 for BO, and 0.81 for STS. Our study is in line with the reported internal consistency, with a Cronbach’s alpha of 0.88 for CS, 0.69 for BO, and 0.85 for STS.

Levene’s test results showed equal variances between the two groups for each target variable. As shown in Table 2, compared to G2, G1 demonstrated significantly different values in terms of the avoidance strategies (AS) dimension of the COPE-NVI scale.

Although, the two groups were significantly different in terms of gender proportion (χ^2^(1) = 4.35; *p*-value = 0.04), no significant differences in the test scores between G1 and G2 emerged.

### Subgroups Analysis Results

As shown in Table 3, the analysis of the subgroups revealed significant differences in the TR (turning to religion) dimension for both of the main groups (G1: *p* < 0.001, G2: *p* = 0.02). Furthermore, we found significant differences in the CS (*p* = 0.04) and BO (*p* = 0.04) dimensions between the two subgroups of G1. The differences found for the other constructs were not statistically significant, but can still provide an overview of the quality of life and on the coping strategies most used by workers with different roles (R1 and R2) in the neurological rehabilitation unit (G2) and the cardiovascular intensive care unit (G1). In this latter setting, the R1 group scored higher for social support (SS) and burnout (BO), while R2 attained higher scores for avoidance strategies (AS), positive attitude (PA), turning to religion (TR), and compassion satisfaction (CS). The values for STS were the same between the two groups. As regards the neurological rehabilitation unit, the R1 subgroup obtained higher scores for problem solving (PO), and R2 for AS, TR, BO, and STS. The SS, PA, and CS values were the same between the two groups.

Furthermore, although the differences in the other constructs were not statistically significant, the response trend may be informative regarding the quality of life, coping styles, and strategies most used in the cardiovascular intensive care unit (G1) and the neurological rehabilitation unit (G2). In this regard, it would appear that healthcare workers belonging to G1 (in addition to adopting AS) are tendentially more likely to seek social support (SS) and have higher levels of compassion satisfaction (CS). On the other hand, G2 health workers have more positive attitudes (PA), but even higher values of burnout (BO) and secondary stress trauma (STS). The mean values obtained for the problem solving (PS) and turning to religion (TR) scales were the same in the two groups.

## 4. Discussions

The cardiovascular intensive care unit is an intensive and sub-intensive care unit in a hospital that is specialized in the clinical management of patients suffering from acute coronary syndrome or severe life-threatening cardiological pathologies. Within this context, having to continually deal with the life-and-death struggle of many patients may be perceived as too much of an emotional burden to handle. This could lead to the emergence of avoidance strategies and the active search for social support. From another point of view, however, being able to cope with these highly stressful situations can confer a great sense of agency over the management and course of the disease. This has a positive influence on the job satisfaction derived from carrying out one’s care work. A rehabilitation ward such as the one considered in this study constitutes a very complex environment. In fact, patients’ health problems can become multiple, and they often turn chronic depending on the severity of the illness. Health professionals working within this context may perceive a lack of effectiveness that compromises their job satisfaction.

Within the cardiovascular intensive care unit, the R1 subgroup scored the same as R2 for secondary traumatic stress (STS), but obtained higher values for the social support (SS) and burnout (BO) scales. Burnout has been described as a prolonged response to chronic emotional and interpersonal stress on the job that is often a result of a period of expending excessive effort at work while having too little recovery time [46,47]. Healthcare workers operating in highly stressful medical environments, particularly in intensive care units (ICUs), may exhibit increased vulnerability to burnout [48,49]. This susceptibility can be attributed to their daily exposure to challenging situations involving death and pain care. Moreover, ICU personnel can experience heightened stress levels due to the significant morbidity and mortality rates they encounter, along with ethical dilemmas [50]. Additionally, various other factors are associated with burnout, including age, sex, marital status, personality traits, ICU work experience, work environment, workload, shift work, and involvement in end-of-life decision-making [51]. Poor physical/mental health and subjective/psychological wellbeing are also associated with high levels of burnout. Indeed, high levels of emotional exhaustion and depersonalization, as well as low levels of personal accomplishment, negatively affect one’s subjective and psychological well-being [52] and often lead to the development of depressive symptoms [53]. Moreover, these healthcare workers are constantly reminded of death and, therefore, of their own mortality, and this makes them susceptible to death anxiety [54]. These results contradict those found in other studies in the literature, which have identified higher levels of burnout for nurses [55,56], especially for those working night shifts [57,58,59]. In this study, the higher burnout values obtained by R1 could also be related to issues of personnel management and to interpersonal relationships, which are identified as one of the main factors that can cause burnout syndrome [60]. Burnout causes a deterioration in quality of care, increasing the risk of mortality in patients due to poor performance and errors in the healthcare environment. Future work should address the effective management of the factors negatively affecting ICU professionals.

On the other hand, the scores obtained by the R2 subgroup were the same as those of R1 for social support (SS), positive attitude (PA), and compassion satisfaction (CS), but were higher for positive attitude (PA), problem solving (PS), compassion satisfaction (CS), and turning to religion (TR). The most common coping strategies that the studies in the literature have shown were efficient, in particular social and emotional support, physical activity, physical self-care, and emotional and physical distancing from work. The coping mechanisms associated with lower rates of burnout were physical well-being, clinical variety, setting boundaries, religious faith, passion for one’s work, realistic expectations, remembering patients, and organizational activities. Among the adaptive coping strategies, in particular, a relationship between religiosity and the burnout dimensions was observed [61]. The presence of religious beliefs can help workers to handle problems at work and can make healthcare more satisfying [62].

Within the neurorehabilitation department, the high scores obtained by R2 for burnout (BO) and secondary traumatic stress (STS) could be related to the chronicity of many patients and the perception that the intensity of one’s efforts is not proportional to an improvement in the patient’s health. An interesting fact involves the scores obtained for the turning to religion (TR) scale. Religion and spirituality are integral aspects of one’s self-identity [63]. Many healthcare workers hold religious beliefs or believe in a God [64,65], often using this to guide important personal decision making. It would seem that religiosity and spirituality are resources that can foster adaptability within such complex organizational contexts [66,67]. They constitute a coping strategy for mental illness, especially in highly stressful contexts [68,69,70,71,72].

There is substantial evidence that spiritual well-being is an important determinant of overall health, longevity, and quality of life [73]. It even seems that religion/spirituality influence the practice of medicine and communication between health workers and patients, reducing their emotional distress and upholding hope [74]. A study of Prazeres et al. [75] found that healthcare workers with higher levels of religiosity showed less pandemic-related anxiety. One might also think of including this as a construct within an intervention hypothesis with respect to cultural and educational factors in order to enhance its effects. Oxhandler and Parrish [76] stated that the willingness to include religiosity within clinical practice does not differ significantly between different professionals who are part of the healthcare workforce. Cunha et al. [77] suggest that religion, religiosity, and spirituality should be incorporated into nurse training in order to improve comprehension and competence in these areas of practice. On the other hand, over-reliance on a higher will or a divine plan that is not dependent on the person could lead to excessive passivity in facing and handling the problems of everyday life. This is corroborated in the literature, where it has been shown that individuals with religious beliefs use avoidance strategies more frequently than non-believers [78]. Another interesting finding to discuss is that that the values obtained from R2 were higher for avoidance strategies (AS) and turning to religion (TR) constructs in both G1 and G2, which would suggest that this is a pattern that is more related to the work task than to the work environment.

Compared to the cardiovascular intensive care unit (G1), in the neurological rehabilitation unit (G2), R1 appears to be more problem-oriented and to have a more positive attitude and religious orientation. However, it makes less use of social support and avoidance strategies. This pattern in G2 is combined with lower levels of compassion satisfaction (CS), burnout (BO), and secondary traumatic stress (STS) than in G1.

The R2 subgroup in G2 demonstrated a less positive attitude, sought less social support, and made less use of avoidance strategies than that in G1. Problem solving and religious orientation were also lower in this ward. This pattern correlates with a lower CS and higher BO and STS values than in G1. Professionals regularly exposed to the traumatic experiences of patients are particularly susceptible to developing high levels of BO and STS [79,80,81,82].

The wellbeing of healthcare workers is fundamental for the effective function of healthcare performances and systems. Most healthcare professionals conceptualize wellbeing as absence of stress rather than as a positive state [83].

High levels of BO and STS are associated with exhaustion, irritability, anger, and negative coping behaviors, including alcohol and drug abuse.

High and sustained levels of stress have been implicated in various health consequences, including cardiovascular disease, increased susceptibility to infections, physiological disorders, and mental illnesses, which can significantly impact the job performance of healthcare professionals. Burnout (BO) and secondary traumatic stress (STS) are known to contribute to higher absenteeism rates and a diminished sense of enjoyment and satisfaction with work, as well as reduced levels of sympathy and empathy [84].

Physiologically, acute stress leads to increased heart rate, systolic and diastolic blood pressure, and levels of salivary interleukin-1β [85]. Several stress-related biomarkers are also affected, such as decreased leptin levels, increased ghrelin levels [86], and elevated salivary levels of α-amylase and dehydroepiandrosterone [87]. These physiological responses highlight the impact of stress on the body and its potential implications for overall health and well-being.

Among the limitations of this study is the small sample taken into consideration. In addition, due to our inability to access this information, it was not possible to determine the total number of healthcare workers working in the hospitals under study. Although this is an observational study, this resulted in the impossibility of establishing representativeness with our sample. In future research, it may be useful to attempt to address these two issues.

## 5. Conclusions

This work highlights the importance and necessity of introducing the assessment of coping strategies and quality of life within work contexts into A&F protocols. Monitoring the psychological conditions of healthcare workers would allow for the implementation of targeted prevention and intervention programs [88]. These interventions and programs could become an integral part of health policies so as to prevent the health-related and economic consequences of the resulting disabling physical and mental health outcomes [27]. Furthermore, an investigation of the coping strategies employed would make it possible to identify fragilities within the team and to prevent psychological suffering, especially in contexts where working conditions are stressful. Attention to the physical, psychological, and emotional well-being of healthcare workers is, therefore, a fundamental step in the implementation of a process of improving care services such as A&F.

## Figures and Tables

**Table 1:** Description of the study population.

	G1	G2	All
**Subjects**	49	46	95
Age *(months)*	47.7 ± 11.7	46.3 ± 8.8	47 ± 10.4
**Gender**			
Male	24 (49.0)	12 (26.1)	36 (37.9)
Female	25 (51.0)	34 (73.9)	59 (62.1)
**Subgroups**			
R1	23	18	41
R2	26	28	54

Mean ± standard deviation was used to describe continuous variables; proportions (numbers and percentages) were used to describe categorical variables. Legend: G1 = Group 1, G2 = Group 2; R1 = Role 1, R2 = Role 2.

**Table 2:** Statistical comparisons of clinical scores between the two groups of participants.

Assessment	G1	G2	*p*-Value
*COPE-NVI*			
** SS **	30.0 (25.0–33.0)	26.0 (23.0–33.8)	0.24
** AS **	24.0 (21.0–27.0)	21.0 (18.3–24.0)	**<0.001**
** PA **	33.0 (30.0–38.0)	33.5 (29.3–36.8)	0.88
** PS **	34.0 (30.0–38.0)	34.0 (30.0–37.0)	0.98
** TR **	21.0 (18.0–27.0)	21.0 (18.3–24.0)	0.77
*ProQOL*			
** CS **	43.0 (38.0–47.0)	40.5 (35.0–45.8)	0.14
** BO **	21.0 (18.0–26.0)	23.0 (19.3–28.5)	0.20
** STS **	19.0 (15.0–23.0)	20.5 (16.3–26.8)	0.22

Scores are expressed as medians (first–third quartile); significant differences are in bold. Legend: COPE-NVI dimensions: SS = social support, AS = avoidance strategies, PA = positive attitude, PS = problem solving, TR = turning to religion; ProQOL dimensions: CS = compassion satisfaction, BO = burnout, STS = secondary traumatic stress; G1 = Group 1, G2 = Group 2.

**Table 3:** Statistical comparisons of clinical scores between the role subgroups for each of the two groups of participants.

	Assessment	R1	R2	*p*-Value
**G1**	*COPE-NVI*			
	SS	31.0 (26.0–33.5)	26.5 (24.0–31.5)	0.11
	AS	23.0 (20.0–27.0)	25.5 (22.0–27.0)	0.34
	PA	32.0 (29.0–35.0)	34.0 (31.3–38.8)	0.10
	PS	32.0 (28.5–37.5)	35.0 (32.3–37.8)	0.18
	TR	19.0 (17.0–21.0)	25.5 (20.3–27.8)	**<0.001**
	*ProQOL*			
	CS	41.0 (34.5–46.0)	45.0 (41.3–48.0)	**0.04**
	BO	23.0 (20.0–27.0)	20.5 (17.3–22.8)	**0.04**
	STS	19.0 (16.0–23.5)	19.0 (15.3–23.0)	0.80
**G2**	*COPE-NVI*			
	SS	26.0 (23.3–34.0)	26.0 (23.0–33.0)	0.67
	AS	20.0 (18.0–22.0)	21.0 (19.0–25.3)	0.38
	PA	33.5 (29.3–35.8)	33.5 (29.8–37.3)	0.56
	PS	34.5 (30.5–37.8)	33.0 (29.5–37.0)	0.44
	TR	19.5 (18.0–21.0)	24.0 (19.8–27.0)	**0.02**
	*ProQOL*			
	CS	40.5 (38.0–45.0)	40.5 (33.8–46.0)	0.93
	BO	22.0 (20.3–24.5)	23.5 (19.0–29.0)	0.60
	STS	18.5 (15.3–23.8)	22.0 (17.0–29.0)	0.18

Scores are in median (first–third quartile); significant differences are in bold. Legend: COPE-NVI dimensions: SS = social support, AS = avoidance strategies, PA = positive attitude, PS = problem solving, TR = turning to religion; ProQOL dimensions: CS = compassion satisfaction, BO = burnout, STS = secondary traumatic stress; G1 = Group 1, G2 = Group 2; R1 = Role 1, R2 = Role 2.

## Data Availability

Data are available from the authors upon reasonable request and with permission from the IRCCS Centro Neurolesi Bonino-Pulejo.
